# Iodoform Substitutes

**Published:** 1898-04

**Authors:** 


### Abstracts and Translations.

### IODOFORM SUBSTITUTES.

IODOFORM contains about twenty-nine parts of pure iodine in thirty. Its antiseptic and deodorizing effect is, therefore, due to this element; the carbon and hydrogen with which it is associated render the iodine non-irritant, either when taken by the mouth or applied topically. A great disadvantage attending the use of iodoform is its disagreeable odor. It is impossible to entirely mask this odor, although it may be covered to a great extent by mixing it with various aromatic substances, such as balsam of Peru, Tonquin bean, coumarin, menthol, thymol, oil of sassafras, attar of roses, oil of peppermint, oil of anise, oil of eucalyptus, carbolic acid, etc. A number of iodoform substitutes have been introduced, some containing iodine, and hence supposed to act like iodoform, and others with no iodine in their composition, but which have a similar action to iodoform. Many of these substitutes are proprietary articles of German origin. The results of inquiries made at hospitals, of pharmacists, and of wholesale chemists and druggists, show that these iodoform substitutes have in no way diminished the use of iodoform, and that, in fact, they are in very small demand.

Iodol (tetra-iodo-pyrrol) stands at the head of the list of iodoform substitutes as regards the amount of iodine present. It contains about twenty-seven parts in thirty. Iodol is obtained by precipitating pyrrol with iodo-iodate of potassium. It is a micro-crystalline, brownish-white powder, having a faint thyme-like smell, and is soluble in six parts of absolute alcohol, but nearly insoluble in water. It is said to produce no toxic action like iodoform when wounds are dressed with it, and its application is painless. Iodol has been used with good results in granular and chronic conjunctivitis, hard and soft chancres, and various ulcers much improve under its use. It possesses some anæsthetic action, and acts as an astringent when discharge is copious.

Losophan (meta-tri-iodo-cresol) contains twenty-four parts of pure iodine in thirty. It is a grayish crystalline powder, soluble in alcohol, chloroform, oils, and fats. It has been found useful in parasite skin-affections, but not of general value, and it is apt to cause irritation.

Iodo-salicylic acid and di-iodo-salicylic acid are iodine compounds of salicylic acid in which one or two atoms of hydrogen respectively are replaced by iodine. Di-iodo-salicylic acid contains twenty parts of iodine in thirty, iodo-salicylic acid fifteen in thirty. These compounds are powerful antiseptics. They possess the combined action of iodine and salicylic acid, and have been successful in the treatment of acute polyarticular rheumatism where salicylates have failed. These acids are in the form of white micro-crystalline powders, slightly soluble in water, soluble in alcohol, ether, fixed oils, and, like salicylic acid, also in collodion.

Sozoiodol (di-iodo-para-phenolsulphonic acid) is composed of fifty-four per cent. iodine, seven per cent. sulphur, and twenty per cent. phenol. It has been combined with sodium, potassium, ammonium, lead, mercury, and zinc, which have been suggested as odorless substitutes for iodoform. The sodium salt which has been used is in colorless shining acicular crystals, soluble in water. The salt is well tolerated as an external application. It has been given internally in doses of twenty grains three times a day. Sozoiodol has been found useful in the treatment of whooping-cough,—three grains blown into each nostril once daily. A solution of sozoiodol-mercury with iodide of sodium has been recommended for intramuscular injection in syphilis.

Aristol (di-thymol-iodide) is a reddish-brown powder containing 45.8 per cent. of iodine. It is insoluble in water, glycerin, or alcohol, but soluble in ether or oils. It has been used successfully in various skin-affections, psoriasis, eczema, rhinitis, ozæna, and lupus, but has proved unsatisfactory in lichen rubra, soft chancre, and gonorrhœa. Aristol has a certain effect on venereal ulcers, but acts very slowly; the only advantage it possesses over iodoform is absence of smell; its activity is inferior. It has been found of service in the first and second stages of pulmonary tuberculosis when no cavities exist. It also lessens cough and night-sweats. Burns and scalds have been successfully treated with aristol, and the application in a powder to the cornea in keratitis and in an ointment in corneal ulcers has given good results. It is of great value in nasal affections; it lessens the discharge, relieves pain, and stops bleeding when used as an insufflation in cancer of cervix uteri.

Europhen (iso-butyl-ortho-cresyl-iodide) occurs as a pale-orange, non-crystalline powder, containing twenty-eight per cent. of iodine. It possesses powerful antiseptic properties, and, being resinous to the touch, it adheres well to mucous membrane and wound surface, and does not easily cake. A given weight as compared with iodoform will cover a surface five times the area of the latter. It is non-poisonous, and acts only when brought into contact with secreting surfaces, which decompose it and liberate iodine. Its lightness and freedom from odor make it especially useful in dentistry. The general opinion of europhen is that it may be used with advantage in all cases where iodoform has been used. Improvement has followed its use by inunction and subcutaneous injection in tubercular leprosy, and it has been found serviceable in eye-diseases, otitis, and ozæna. Europhen has failed in eczema, psoriasis, and gonorrhœa, but has given satisfactory results in simple and venereal ulcers, and in oily solution injected daily for syphilis.

Loretin (meta-iodo-ortho-oxy-chinolin-ana-sulphonic acid) is a bright-yellow, crystalline powder, odorless, and similar in appearance to iodoform. It is very slightly soluble in water or alcohol, and insoluble in ether, but forms soluble salts with alkalies, except with lime. It is non-poisonous and unirritating, and has been used with marked curative effect on burns, ulcers, and other wounds.

Airol, a gallate of bismuth and iodine, is a light grayish-green powder, stable in dry air, but, when left in contact with moisture, iodine is gradually liberated. It is insoluble in water, alcohol, and ether. Airol is astringent and desiccative, as well as being antiseptic.

Di-iodoform (ethylene periodide) occurs in yellow crystals, almost inodorous, insoluble in water, soluble in chloroform, and slightly in alcohol and ether. It is partly decomposed by light. It has been recommended as an antiseptic in place of iodoform.

Antiseptol (iodosulphate of cinchonine) is an odorous brown powder, which has been recommended as a substitute for iodoform. It contains half its weight of iodine, and is soluble in alcohol or chloroform, but is insoluble in water.

The chief non-iodine compounds which have been introduced to compete with iodoform as an antiseptic are dermatol, thioform, and thioresorcin.

Dermatol as a basic gallate of bismuth is recommended as a powerful non-irritant antiseptic and desiccant. Applied to wounds, it induces rapid cicatrization, does not irritate, nor give rise to toxic effects. It is not well suited to septic wounds, and insufficiently stimulating in chronic indolent ulcers. It is a quicker microbicide than iodoform. Its use in the treatment of venereal ulcers has been successful, and also in pustular and diphtherial conjunctivitis, corneal ulcers, and pannus, but of little use in blepharitis. Dermatol is a yellow powder, odorless, and insoluble in water.

Thioform, a basic bismuth salt of di-thio-salicylic acid, is a yellowish-brown powder, odorless, and insoluble in water. Its claim to supplant iodoform is based upon its freedom both from odor and from toxic properties, its greater antiseptic strength, and its desiccative action. It freely absorbs secretions from wounds without forming a crust. As a desiccant antiseptic, especially for eye cases, it has been recommended.

Thioresorcin is a combination of sulphur with resorcin. It is a yellowish-white, inodorous, and non-toxic powder, insoluble in water, slightly so in alcohol and ether. As a dusting-powder, it has been used instead of iodoform, and a ten- to twenty-per-cent. ointment for eczema, psoriasis, and other skin-diseases.—*British Medical Journal.*

